# Decoding Pancreatic Neuroendocrine Tumors: Molecular Profiles, Biomarkers, and Pathways to Personalized Therapy

**DOI:** 10.3390/ijms26167814

**Published:** 2025-08-13

**Authors:** Linda Galasso, Federica Vitale, Gabriele Giansanti, Giorgio Esposto, Raffaele Borriello, Irene Mignini, Alberto Nicoletti, Lorenzo Zileri Dal Verme, Antonio Gasbarrini, Maria Elena Ainora, Maria Assunta Zocco

**Affiliations:** Department of Internal Medicine and Gastroenterology, Fondazione Policlinico Universitario Agostino, Gemelli IRCCS, Catholic University of Rome, 00168 Rome, Italy; linda.galasso@guest.policlinicogemelli.it (L.G.); federica.vitale@guest.policlinicogemelli.it (F.V.); gabriele.giansanti01@icatt.it (G.G.); giorgio.esposto@guest.policlinicogemelli.it (G.E.); raffaele.borriello01@icatt.it (R.B.); irene.mignini@guest.policlinicogemelli.it (I.M.); alberto.nicoletti@guest.policlinicogemelli.it (A.N.); lorenzo.zileridalverme@policlinicogemelli.it (L.Z.D.V.); antonio.gasbarrini@policlinicogemelli.it (A.G.); mariaassunta.zocco@policlinicogemelli.it (M.A.Z.)

**Keywords:** pancreatic neuroendocrine tumors, menin 1, death domain-associated protein, alpha thalassemia/mental retardation syndrome X-linked

## Abstract

Pancreatic neuroendocrine tumors (pNETs) are rare malignancies, accounting for 1–2% of pancreatic cancers, with an incidence of ≤1 case per 100,000 individuals annually. Originating from pancreatic endocrine cells, pNETs display significant clinical and biological heterogeneity. Traditional classification based on proliferative grading does not fully capture the complex mechanisms involved, such as oxidative stress, mitochondrial dysfunction, and tumor-associated macrophage infiltration. Recent advances in molecular profiling have revealed key oncogenic drivers, including MEN1 (menin 1), DAXX (death domain–associated protein), ATRX (alpha thalassemia/mental retardation syndrome X-linked), CDKN1B (cyclin-dependent kinase inhibitor 1B) mutations, chromatin remodeling defects, and dysregulation of the mTOR pathway. Somatostatin receptors, particularly SSTR2, play a central role in tumor biology and serve as important prognostic markers, enabling the use of advanced diagnostic imaging (e.g., Gallium-68 DOTATATE PET/CT) and targeted therapies like somatostatin analogs and peptide receptor radionuclide therapy (PRRT). Established biomarkers such as Chromogranin A and the Ki-67 proliferation index remain vital for diagnosis and prognosis, while emerging markers, like circulating tumor DNA and microRNAs, show promise for enhancing disease monitoring and diagnostic accuracy. This review summarizes the molecular landscape of pNETs and highlights genomic, transcriptomic, proteomic, and epigenomic factors that support the identification of novel diagnostic, prognostic, and therapeutic biomarkers, ultimately advancing personalized treatment strategies.

## 1. Introduction

Pancreatic neuroendocrine tumors (pNETs) constitute a rare and heterogeneous subset of neoplasms representing less than 2% of all pancreatic cancers [1]. Despite their low prevalence, the incidence of pNETs has shown a gradual increase in recent decades, particularly in Europe. However, the available epidemiological data remains limited and largely retrospective, often derived from national or regional registries with variable methodological rigor [2]. Data from the SEER program (1973–2012) indicate that pNETs comprised approximately 23% of 6291 reported gastroenteropancreatic neuroendocrine carcinoma (GEP-NEC) cases, with incidence rising from 1.5 to 4.6 per million over 40 years, likely reflecting both improved detection and a true increase in occurrence [3]. pNETs are classified as either functional or non-functional based on their hormone production, which is responsible for the symptoms experienced by patients. However, non-functional pNETs are more common, accounting for 50–85% of cases [2]. Risk factors include a family history of cancer, tobacco use, diabetes, and alcohol consumption. Among GEP-NETs, pancreatic tumors have the worst prognosis, with a median overall survival of 3.6 years, often due to their incidental detection or diagnosis at advanced stages [4]. Immunohistochemically, pNETs typically express markers such as chromogranin A, synaptophysin, and neuron-specific enolase. Their clinical course is variable, with approximately one-third of patients presenting with metastatic disease at diagnosis. Grading is currently based on mitotic count and the Ki-67 proliferation index, as outlined in the 2023 European Neuroendocrine Tumor Society (ENETS) guidelines. The Ki-67 index thresholds distinguish G1 (<3%), G2 (3–20%), and G3 (>20%) tumors [5]. Despite its central role in diagnostic and therapeutic decisions, the Ki-67 assessment is hampered by variability in methodology, inter-observer differences, and technical limitations [6,7,8]. Moreover, tumors with the same grade can exhibit markedly different behaviors, highlighting the need for molecular characterization to improve prognostic accuracy and guide treatment. Advances in high-throughput sequencing have revealed frequent alterations in genes such as MEN1 (menin-1) death domain-associated protein (DAXX), and alpha thalassemia/mental retardation syndrome X-linked (ATRX) as well as pathways involved in chromatin remodeling, mTOR (mechanistic target of rapamycin) signaling, DNA repair, and telomere maintenance. Notably, many sporadic pNETs harbor germline mutations, particularly in DNA repair genes, with potential implications for screening and therapeutic strategies [9,10,11]. While histology-based classification remains standard, molecular profiling may offer a more precise understanding of tumor biology and treatment response. Currently, surgical resection is the only potentially curative option for localized pNETs, while advanced cases are treated with somatostatin analogs, peptide receptor radionuclide therapy (PRRT), everolimus, sunitinib, or alkylating agent-based chemotherapy [2,12].

This review aims to provide an updated overview of the molecular mechanisms driving pNET development, identify promising diagnostic and prognostic biomarkers, and examine the current and emerging personalized therapeutic strategies.

## 2. Molecular Landscape and Evolving Insights into Pancreatic Neuroendocrine Tumors

### 2.1. Genomic and Epigenetic Alterations: Key Drivers of pNETs Pathogenesis

pNETs exhibit considerable molecular heterogeneity, characterized by a relatively low overall mutational burden but recurrent alterations in genes involved in chromatin remodeling, cell cycle regulation, and intracellular signaling. Genomic and epigenomic profiling has identified frequent somatic mutations in a limited number of key genes, most notably MEN1, DAXX, and ATRX [9,10,11]. MEN1, the most mutated gene in sporadic pNETs, encodes the tumor suppressor menin, which plays a central role in transcriptional regulation, genomic integrity maintenance, and histone modification [13]. Loss-of-function mutations in MEN1 are found in approximately 35–45% of cases and are implicated in both hereditary and sporadic forms of the disease. Menin’s interaction with histone methyltransferase complexes suggests that its inactivation disrupts epigenetic regulation, leading to aberrant chromatin remodeling and the impaired expression of genes critical for endocrine cell differentiation [14].

Mutations in DAXX and ATRX occur in about 20–25% of pNETs, are usually mutually exclusive, and are strongly associated with the alternative lengthening of telomere (ALT) phenotype, a telomerase-independent telomere maintenance mechanism linked to genomic instability. Loss of nuclear expression of DAXX or ATRX correlates with ALT activation and has been proposed as a prognostic marker, although its predictive value for treatment response remains unclear [15,16]. Tumors retaining DAXX and ATRX functions often display a more differentiated phenotype and lower proliferation rates, whereas loss of their expression and ALT activation are associated with an increased risk of recurrence and metastasis, even without overt histological dedifferentiation [17,18]. In addition to chromatin remodeling, frequent alterations affect the PI3K/AKT/mTOR pathway through inactivation of TSC1 (Tuberous sclerosis complex 1), TSC2 (Tuberous sclerosis complex 2), or PTEN (phosphatase and tensin homolog), resulting in a constitutive activation of mTOR complex 1 (mTORC1) and promoting tumor cell growth, survival, and metabolic reprogramming [19,20,21]. Unlike pancreatic ductal adenocarcinoma (PDAC), pNETs rarely harbor mutations in classical oncogenes such as KRAS, BRAF, or PIK3CA. However, high-grade, poorly differentiated neuroendocrine carcinomas, molecularly and clinically distinct from well-differentiated pNETs, frequently exhibit mutations in tumor suppressors TP53 and RB1, a higher mutational load, and genomic instability, underscoring their classification as a separate entity [22,23].

### 2.2. From Genomics to Multi-Omics: Unraveling Complexity and Therapeutic Opportunities in pNETs

While early genomic and epigenetic investigations identified the core molecular alterations and broad subtypes of pNETs, the integration of multi-omics and single-cell technologies has markedly refined our understanding of their biology. This comprehensive approach has illuminated the complexity of tumor heterogeneity, revealed novel mechanisms of therapeutic resistance, and facilitated the development of more precise molecular classifications, key steps toward effective precision oncology in this challenging malignancy. Genomic, transcriptomic, proteomic, and epigenomic data have collectively uncovered distinct biological subtypes of pNETs. Transcriptomic and proteomic analyses, for instance, have delineated lineage-specific groups, such as the PDX1-high (beta cell-like) subtype, typically associated with insulinomas, low proliferative activity, and favorable outcomes, and the ARX-high (alpha cell-like) subtype, characterized by frequent MEN1, DAXX, and ATRX mutations, and linked to non-functioning tumors with poorer prognoses [24,25,26]. Additional subtypes, including a metastasis-like primary cluster and a proliferative group, exhibit features like stemness, hypoxia response, and heightened cell cycle activity, all correlating with adverse clinical behavior. These transcriptomic distinctions have been substantiated by proteomic profiling, which showed differential enrichment of mitochondrial metabolism and DNA replication pathways [26]. Epigenomic studies have provided a complementary layer of insight, identifying three major DNA methylation patterns, beta-like, alpha-like, and intermediate. The beta-like subtype is marked by PDX1 hypomethylation, the absence of ALT, and a better prognosis; the alpha-like subtype displays ARX expression, recurrent MEN1 mutations, and fewer chromosomal aberrations. In contrast, the intermediate group, defined by DAXX/ATRX mutations and ALT positivity, shows increased genomic instability, reduced MGMT expression, and a hybrid methylation profile, suggesting potential sensitivity to alkylating agents like temozolomide [27,28,29,30]. By integrating these layers, multi-omic approaches have enabled a more cohesive and functional characterization of pNETs, bridging molecular subtypes with clinical relevance. Studies combining genomic, transcriptomic, proteomic, and miRNA analyses have revealed ATM-dependent signaling, modulated both genetically and epigenetically through microRNAs, as a possible mediator of therapy resistance [31,32,33,34]. Moreover, circulating microRNAs have emerged as accessible and minimally invasive biomarkers for diagnosis, prognosis, and patient stratification. Adding yet another dimension, single-cell RNA sequencing (scRNA-seq) has unveiled intratumoral heterogeneity and previously unrecognized tumor epithelial subpopulations. Notably, Zhou et al. identified discrete gene expression programs related to proliferation (CDK, CDKN1, E2F), drug resistance (DHFR, TOP2A, BCL2), and immune escape, and proposed a metastatic risk signature based on PCSK1 and SMOC1 co-expression [35]. In parallel, Hoffman et al. highlighted the sparse expression of classical immune checkpoint molecules (PDL1, PDL2, HLA-G, LGALS9), indicating likely resistance to current immune checkpoint inhibitors and supporting the need for alternative immunotherapeutic strategies [36]. In summary, the shift from single-layer analyses to integrated multi-omic and single-cell approaches has dramatically enhanced the resolution and interpretability of the molecular landscape in pNETs, uncovering novel biomarkers, clarifying tumor subtypes, and pointing toward new therapeutic avenues.

### 2.3. The Tumor Microenvironment in pNETs: Driver of Progression, Immune Evasion and Therapeutic Resistance

The tumor microenvironment (TME) plays a pivotal role in shaping the pathophysiology, progression, and therapeutic response of pNETs. Far from being passive bystanders, non-neoplastic components of the TME, including metabolic stressors, immune cells, and stromal elements, actively engage in reciprocal interactions with tumor cells, significantly influencing disease evolution and clinical outcomes [9].

A key feature of the pNET microenvironment is oxidative stress, which arises from an imbalance between reactive oxygen species (ROS) production and cellular antioxidant defenses. ROS exert dual roles depending on their concentration: at moderate levels, they act as signaling molecules, activating survival and proliferative pathways such as PI3K/AKT/mTOR and MAPK (mitogen-activated protein kinase) cascades; at high levels, they cause DNA damage, genomic instability, and oxidative modification of cellular macromolecules, thereby fostering tumor heterogeneity and therapeutic resistance [37,38]. Closely linked to oxidative stress is mitochondrial dysfunction, increasingly recognized as a hallmark of neuroendocrine tumor biology [38]. In pNETs, mutations in nuclear-encoded regulators of mitochondrial metabolism, such as TSC1, TSC2, and PTEN, lead to constitutive mTORC1 activation. This promotes anabolic metabolism, enhances mitochondrial biogenesis, and inhibits autophagy, contributing to the accumulation of dysfunctional mitochondria and further amplifying ROS generation [39,40,41].

Metabolic plasticity is another critical adaptive strategy in pNETs. Tumor cells exhibit the capacity to reprogram their metabolism in response to microenvironmental stressors, such as hypoxia, nutrient limitation, and oxidative pressure. This plasticity enables a dynamic shift between oxidative phosphorylation and glycolysis to sustain bioenergetic and biosynthetic demands under adverse conditions, thereby supporting continued tumor growth, survival, and therapy resistance [42]. Importantly, mitochondrial dysfunction extends its influence beyond cellular metabolism by modulating tumor–immune interactions. Mitochondrial-derived ROS and damage-associated molecular patterns (DAMPs) influence the phenotype of tumor-associated macrophages (TAMs), skewing them toward an immunosuppressive, M2-like profile [43,44].

TAMs are key immune constituents of the pNETs’ TME. In pNETs, TAMs typically exhibit an M2-like phenotype, characterized by the secretion of anti-inflammatory cytokines (e.g., IL-10, TGF-β), pro-angiogenic factors (e.g., VEGF), and matrix-remodeling enzymes (e.g., MMP9), all of which contribute to tumor progression, angiogenesis, and immune suppression [45]. High TAM density is associated with increased tumor invasiveness and poorer clinical outcomes. Moreover, TAMs orchestrate a permissive microenvironment by interacting with tumor cells, fibroblasts, endothelial cells, and regulatory T cells, creating a niche that promotes immune evasion and metastatic dissemination [46].

The immune landscape of pNETs is heterogeneous and stage dependent. Well-differentiated (G1–G2) tumors generally exhibit a “cold” immune phenotype with sparse tumor-infiltrating lymphocytes (TILs). In contrast, high-grade (G3) pNETs show increased infiltration of PD-1 (programmed cell death protein 1) high T cells and PD-L1 (programmed death ligand 1) high M2-type macrophages, correlating with higher Ki-67 indices, lymph node metastases, and worse recurrence-free and overall survival [47,48]. pNETs actively secrete immunosuppressive cytokines such as TGF-β, IL-10, and IL-6. These cytokines recruit and expand myeloid-derived suppressor cells (MDSCs) and TAMs, particularly CD68^+^ and M2-like subsets, while inhibiting effector T cell responses and promoting Treg-mediated immunosuppression [49,50]. This immunosuppressive cytokine milieu supports tumor growth, angiogenesis, and resistance to immune-based therapies. Elevated expression of PD-1 on T cells and PD-L1 on TAMs has been linked to poor prognoses and increased tumor aggressiveness, further underscoring the role of dynamic tumor–immune crosstalk in shaping disease behavior [51].

Transcriptomic analyses of metastatic pNETs reveal an increased infiltration of T cells and upregulation of inflammatory signaling pathways, including chemokines such as CCR5. Notably, agents like vorinostat can enhance CCR5 expression, potentially increasing T cell recruitment and modulating the immune microenvironment for therapeutic benefit [52,53].

**Figure 1:**
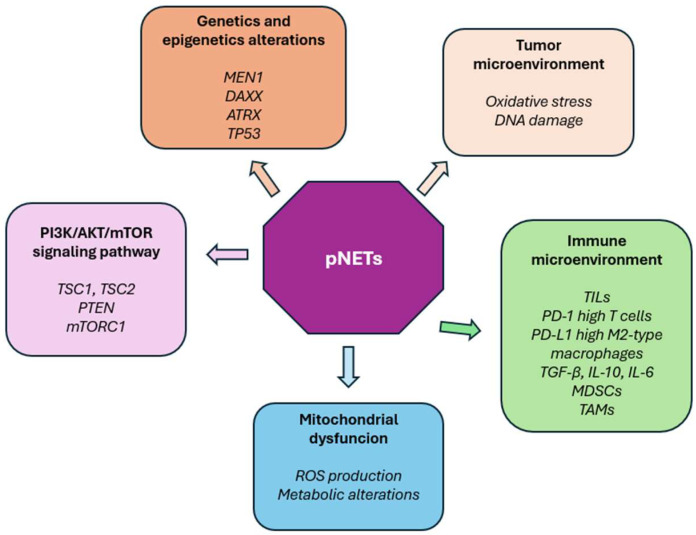
The multifactorial etiopathogenesis of pNETs consists of genetic and epigenetic factors associated with alterations of the tumor microenvironment that act at the molecular level. MEN1: menin 1, DAXX: death domain–associated protein, ATRX: alpha thalassemia/mental retardation syndrome X-linked, TSC1: tuberous sclerosis complex 1, TSC 2: tuberous sclerosis complex 2, TP53: tumor protein p53, PTEN: phosphatase and tensin homolog, mTORC1: mechanistic target of rapamycin complex 1, ROS: reactive oxygen species, TILs: tumor-infiltrating lymphocytes, PD-1: programmed cell death protein 1, PD-L1: programmed death ligand 1, TGF-β: transformin growth factor beta, IL-10: interleukin 10, IL-6: interleukin 6, MDSCs: myeloid-derived suppressor cells, TAMs: tumor-associated macrophages.

Figure 1 depicts the complex interplay between genetic and epigenetic alterations in pNETs and components of the tumor microenvironment.

## 3. Old and New Biomarkers in pNETs: An Integrated Diagnostic Approach

Accurate diagnosis and risk stratification of pNETs rely on a combination of histopathologic, immunohistochemical, and molecular biomarkers. These markers are essential not only for confirming neuroendocrine differentiation but also for assessing tumor aggressiveness and guiding therapeutic decisions.

### 3.1. Established Markers: Chromogranin A, Ki-67 Index

#### 3.1.1. Chromogranin A (CgA) in pNETs: Clinical Utility and Diagnostic Limitations

Although Chromogranin A (CgA), a 439-amino acid glycoprotein encoded by the CHGA gene on chromosome 14q32.12 [54], has long been considered a cornerstone biomarker in the management of pNETs; its clinical utility is increasingly questioned due to several notable limitations. While CgA is secreted alongside catecholamines and other bioactive peptides such as serotonin, insulin, and somatostatin, and its proteolytic fragments (e.g., vasostatin-1, catestatin, and pancreastatin) are involved in neuroendocrine signaling, angiogenesis, and immune regulation [55,56], its actual diagnostic and prognostic reliability is inconsistent. Although CgA expression reflects the secretory activity and granule content of tumor cells, making it a commonly used biomarker for diagnosis, prognosis, and treatment monitoring [57], its sensitivity for detecting early-stage or localized pNETs is suboptimal. In fact, in such cases, sensitivity ranges only between 29.6% and 51.9%, with better performance seen in tumors > 2 cm or metastatic disease, where tumor burden is higher [58]. Meta-analyses and individual studies have reported high specificity (up to 95%) and moderately high sensitivity (73%) for NET diagnosis [59], but these results may overestimate its diagnostic value in clinical practice, especially when CgA is used in isolation. A 2018 study confirmed this discrepancy, reporting a reduced sensitivity (66%) in well-differentiated pNETs [59]. Moreover, Tseng et al. identified significant associations between plasma CgA levels and tumor size (*p* = 0.03), metastatic spread (*p* = 0.02), and disease stage (*p* = 0.03), but again, these correlations emphasize the biomarker’s dependence on tumor burden rather than intrinsic tumor biology [58]. Beyond its modest diagnostic performance, CgA also suffers from a lack of specificity. Elevated levels are not exclusive to neuroendocrine neoplasms and may be observed in several benign or unrelated pathological conditions, including atrophic gastritis, renal or hepatic dysfunction, chronic inflammatory diseases, and in patients taking proton pump inhibitors (PPIs) [60,61,62]. Because of this, PPIs should ideally be discontinued at least two weeks prior to testing [61], a recommendation that is often challenging to implement in routine clinical settings. Compounding these issues is the absence of assay standardization: different commercial kits detect distinct epitopes of the CgA molecule, complicating inter-laboratory comparisons and longitudinal follow-up [63]. This heterogeneity reduces the reliability of CgA for consistent patient monitoring. From a prognostic perspective, although elevated or rising CgA levels have been associated with increased tumor burden, liver metastases, and worse overall survival [64,65,66], findings are not entirely consistent. For instance, the CLARINET trial identified a trend between higher baseline CgA levels and shorter progression-free survival in patients treated with lanreotide, though not all subgroup differences reached statistical significance [67]. Similarly, changes in CgA levels during treatment—particularly with somatostatin analogs or peptide receptor radionuclide therapy (PRRT)—have been proposed as surrogate markers of treatment response, but these remain unreliable and heavily dependent on tumor differentiation and secretory profile [68]. Additionally, in poorly differentiated pancreatic neuroendocrine carcinomas, CgA may be normal or only mildly elevated due to the scarcity of dense-core granules, further limiting its diagnostic relevance in this subgroup [69]. Given these limitations, CgA should not be used as a standalone screening or diagnostic tool. A 2012 meta-analysis strongly recommended combining CgA with imaging and other biochemical tests to improve overall diagnostic accuracy [70]. While CgA remains widely used due to availability and clinical familiarity, the need for more specific, standardized, and reliable biomarkers in the diagnosis and management of pNETs is increasingly evident.

#### 3.1.2. Ki-67 in pNETs: A Valuable but Imperfect Grading Tool

Ki-67, a nuclear protein expressed exclusively during the active phases of the cell cycle and detected by immunohistochemistry using the MIB-1 antibody, is widely considered a cornerstone biomarker for grading pNETs [71,72]. According to the WHO classification, the Ki-67 labeling index stratifies tumors into three grades: G1 (≤2%), G2 (3–20%), and G3 (>20%), correlating, respectively, with indolent, intermediate, and aggressive disease behavior [73,74]. While these categories are broadly accepted and Ki-67 has been validated as a prognostic marker in multiple studies, including a multicenter cohort of 210 pNET patients [75], its clinical application is not without important limitations. First, despite the association between higher Ki-67 indices and reduced survival (with five-year survival dropping from ~85% in G1 to 10–15% in G3 tumors), the intermediate G2 category remains heterogeneous. Subclassification efforts within G2 tumors have demonstrated potential clinical relevance [9,76], but these nuances are not yet reflected in official grading systems, potentially limiting optimal risk stratification in clinical practice. Moreover, while Ki-67 is used to guide treatment decisions, from active surveillance in low-grade tumors to cytotoxic chemotherapy or targeted agents in high-grade disease [77,78,79], its predictive utility is challenged by several technical and biological factors. One major issue is intratumoral heterogeneity, which can lead to sampling bias and underestimation of proliferative activity, particularly in biopsy specimens [80]. This variability risks misclassification of the tumor grade and subsequent undertreatment. In addition, interobserver variability in the manual counting of Ki-67-positive cells is a recognized limitation, often influenced by non-standardized methodology across institutions [81]. Although automated image analysis has been proposed to improve reproducibility, it is not yet widely implemented in routine diagnostic workflows. These technical inconsistencies may compromise the reliability of Ki-67 as a universally applicable tool. Furthermore, the distinction between well-differentiated G3 pNETs and poorly differentiated neuroendocrine carcinomas remains a critical challenge with major therapeutic implications [82]. Despite sharing overlapping Ki-67 indices, these entities differ markedly in clinical behavior, response to therapy, and prognosis, underscoring the need for integrated morphologic and molecular criteria beyond the proliferation index alone. While Ki-67 remains indispensable in the diagnostic and therapeutic algorithm of pNETs, a critical reassessment of its limitations, along with the development of complementary markers and standardized assessment protocols, is essential to refine its role in clinical decision-making.

### 3.2. Emerging Molecular Biomarkers: Circulating Tumor DNA, microRNAs

Non-invasive biomarkers of disease, such as circulating tumor DNA (ctDNA) and circulating microRNAs (miRNAs), represent the most novel diagnostic advance in promising diagnostic approaches for their potential role in improving early diagnosis, prognosis assessment, and therapeutic monitoring in pNETs.

#### 3.2.1. Circulating Tumor DNA in pNETs: Emerging Insights and Challenges for Clinical Implementation

Circulating tumor DNA (ctDNA), consisting of tumor-derived DNA fragments released into the bloodstream primarily through apoptosis, necrosis, and active secretion, has emerged as a promising biomarker with significant potential [83,84]. These fragments harbor comprehensive genetic and epigenetic information, including mutations, copy number variations, methylation patterns, and chromosomal rearrangements. Advances in technologies such as digital droplet PCR (ddPCR), next-generation sequencing (NGS), and targeted deep sequencing have enabled sensitive and specific ctDNA profiling [83,84]. In pNETs, ctDNA analysis represents a novel, minimally invasive approach for tumor genotyping. Experimental studies have identified recurrent mutations in MEN1, ATRX, DAXX, TSC2, and PTEN, with MEN1 and ATRX alterations significantly associated with tumor burden in approximately 40% of cases [85]. Methylation-based ctDNA assays have further demonstrated potential in distinguishing pNETs from other pancreatic lesions with high specificity, as well as in predicting aggressive disease features such as metastatic spread, shorter progression-free survival, and reduced overall survival [32,86]. Notably, dynamic changes in ctDNA levels during treatment have been correlated with therapeutic response, with decreasing ctDNA concentrations reflecting improved clinical outcomes [87]. Compared with traditional biomarkers such as chromogranin A (CgA), ctDNA offers the distinct advantage of directly reflecting the tumor’s molecular landscape, potentially allowing earlier detection of disease progression [87]. A pilot study comparing ctDNA (“liquid biopsy”) with tissue-based biopsy in gastrointestinal and pancreatic NETs reported a consistent pattern of recurrent mutations, supporting the concordance of genomic information between the two modalities [88]. However, it is important to distinguish these encouraging experimental findings from established clinical utility. To date, the application of ctDNA in routine clinical practice remains investigational, primarily due to limitations such as low ctDNA concentrations in early-stage or low-grade pNETs and methodological variability across platforms, which impede standardization and consistent interpretation [89]. Large-scale prospective studies and harmonized analytic protocols are still required to validate ctDNA’s diagnostic and prognostic performance robustly.

#### 3.2.2. MiRNAs in pNETs: Promising Biomarkers with Emerging but Limited Clinical Validation

miRNAs are small, non-coding RNA molecules of approximately 22 nucleotides in length that regulate gene expression post-transcriptionally. They play critical roles in tumor initiation, progression, angiogenesis, and metastasis across various cancers, including pNETS. Their remarkable stability in biological fluids such as serum, plasma, and saliva, often facilitated by encapsulation within exosomes, makes them attractive candidates for clinical biomarker development. Quantification methods like RT-qPCR offer reliable detection, further supporting their translational potential [90,91]. In pNETs, distinct miRNA expression profiles have been observed compared with normal pancreatic tissue and other pancreatic neoplasms such as ductal adenocarcinoma [91]. For example, miR-375 is frequently downregulated, indicating a possible tumor-suppressive function, whereas miR-196a tends to be upregulated in pNETs. Interestingly, miRNAs such as miR-21 and miR-155, which are commonly overexpressed in many solid tumors, display differential or inconsistent expression patterns in pNETs, underscoring the unique molecular landscape of these tumors [92]. Additional key findings include altered expression of miR-7 (implicated in neuroendocrine function), miR-216 and miR-670 (associated with cell adhesion), and miR-129 and miR-670 (linked to metabolic regulation) [91]. Leveraging next-generation sequencing combined with machine learning, a panel comprising miR-106b, miR-130b-3p, miR-127-3p, miR-129-5p, and miR-30d-5p has demonstrated the ability to discriminate between Grade 1 and Grade 2 pNETs with promising sensitivity (83.33%) and specificity (87.5%) [91]. Furthermore, elevated levels of miR-1290 and miR-222 have been associated with high-grade pNETs, while miR-193b and miR-210 correlate with hypoxic response and metastatic propensity. miR-196a2 has emerged as a potential independent prognostic marker indicative of poor survival outcomes [93]. The diagnostic accuracy of miRNAs may be further enhanced by combining their profiles with established biomarkers; for instance, integration of miRNA signatures with chromogranin A (CgA) levels improved diagnostic sensitivity, notably in patients with low baseline CgA [94]. Despite these encouraging experimental results, the clinical applicability of miRNAs in pNETs is currently constrained by a lack of large-scale validation studies and the absence of standardized methodologies for miRNA detection and analysis. These limitations impede the translation of miRNA research into routine diagnostic and prognostic use. Moving forward, systematic efforts to harmonize analytical protocols and conduct multicenter validation trials are essential to unlock the full clinical potential of miRNAs in pNET management.

### 3.3. Somatostatin Receptor 2 (SSTR2) in pNETs: Established Clinical Biomarker with Recognized Strengths and Ongoing Challenges

SSTRs are G-protein-coupled receptors that mediate inhibitory signals affecting hormone secretion and cell proliferation. Among the five identified subtypes (SSTR1–5), SSTR2 is predominantly expressed in well-differentiated pancreatic neuroendocrine tumors (pNETs) and constitutes a cornerstone biomarker for both diagnostic and therapeutic strategies [95,96,97]. The membrane localization of SSTR2 facilitates the application of molecular imaging and targeted radionuclide therapies, which have become integral to current pNET clinical management. High SSTR2 expression provides the biological rationale for using ^68^Ga-labeled somatostatin analogs, such as DOTATATE, in PET/CT imaging, enhancing tumor detection and characterization [98,99,100]. Extensive research supports the diagnostic superiority of ^68^Ga-DOTATATE PET/CT, with sensitivities frequently reported above 90% for lesion detection in well-differentiated NETs. Ambrosini et al.’s meta-analysis involving over 1000 patients confirmed pooled sensitivity and specificity rates of 92% and 85%, respectively, underscoring its clinical reliability [101,102]. Compared with conventional imaging modalities—CT, MRI, or Octreoscan—^68^Ga-DOTATATE PET/CT improves the visualization of small lymph nodes, bone metastases, and occult primary tumors, significantly impacting clinical management in up to 40% of cases [103,104]. This whole-body imaging approach supports accurate staging and individualized treatment planning, influencing surgical decisions or confirming inoperability in as many as 60% of patients [105]. Furthermore, quantitative uptake metrics like SUVmax have shown promising correlations with tumor differentiation, SSTR density, and patient survival [106]. However, despite these strengths, several limitations restrict the universal application of ^68^Ga-DOTATATE PET/CT. Sensitivity diminishes for lesions under 5 mm due to partial volume effects, and false negatives may occur in SSTR-negative tumors. Additionally, false positives can arise from inflammatory processes, complicating interpretation. Practical challenges include variability in SUV measurements across laboratories and logistical constraints posed by the short half-life of Gallium-68, necessitating on-site generator availability [107]. Addressing these issues through improved imaging protocols, standardization efforts, and technological advances will be crucial to further enhance the clinical utility of SSTR2-targeted imaging and therapy in pNETs.

Table 1 and Table 2 provide an overview of the readiness and challenges of biomarkers for clinical application in pNETs.

## 4. Therapeutic Implications and Personalized Approaches of pNETs

The therapeutic landscape of pNETs has expanded considerably, yet challenges remain in optimizing personalized treatment strategies. Advances in tumor biology, receptor expression, and molecular profiling have laid the groundwork for tailored approaches, but translating these insights into clinical practice demands a nuanced understanding of treatment selection, sequencing, and combination.

### 4.1. Somatostatin Analogs (SSAs): Established Foundations and Limitations

SSAs, such as octreotide LAR and lanreotide autogel, are foundational in managing well-differentiated, low- to intermediate-grade pNETs, particularly functional tumors with hormone secretion [108,110]. Large, randomized trials like PROMID and CLARINET have demonstrated significant improvements in progression-free survival with favorable tolerability profiles [67,111]. These results support SSAs as first-line agents for disease stabilization and symptom control. However, the utility of SSAs is largely confined to tumors expressing SSTRs, and their antiproliferative effects, while meaningful, rarely induce tumor regression. Emerging data suggest possible adjuvant benefits, post-resection, in preventing recurrence [112,113], but more robust evidence is needed before routine adoption. Therefore, SSAs serve as a baseline therapy, yet their role in combination regimens and treatment sequencing requires further clarification.

### 4.2. Peptide Receptor Radionuclide Therapy (PRRT): A Paradigm Shift with Strategic Challanges

Although SSAs are frequently employed as monotherapy, their integration with PRRT and other targeted agents is gaining momentum, with combination approaches promising enhanced therapeutic efficacy. SSAs are generally well tolerated, exhibiting minimal systemic toxicity, and remain first-line agents for symptomatic control and disease stabilization in metastatic or unresectable pNETs, demonstrating confirmed somatostatin receptor positivity. For patients with progressive disease or substantial tumor burden, PRRT utilizing radiolabeled SSAs such as ^177Lu-DOTATATE represents a substantial therapeutic advancement [114]. PRRT represents a significant advancement in targeted therapy, leveraging SSTR2 overexpression to deliver cytotoxic beta radiation selectively [114,115]. The NETTER-1 trial initially demonstrated efficacy in midgut NETs, with subsequent studies confirming clinical benefits in pNETs, particularly in patients with high ^68Ga-DOTATATE PET/CT uptake and preserved organ function [116]. Outcomes include objective response rates of up to 60%, a median PFS of 30–34 months, and durable disease control (>70%), often outperforming targeted agents like everolimus and sunitinib in head-to-head comparisons [117]. Nevertheless, PRRT’s full potential is tempered by limitations including reduced sensitivity for small lesions, SSTR-negative tumor subsets, and logistical challenges related to radionuclide availability [107].

Emerging evidence advocates for combining PRRT with other modalities such as stereotactic body radiotherapy (SBRT) and radiosensitizers (capecitabine, temozolomide), aiming to improve efficacy and overcome resistance [118,119].

Additionally, alpha-emitters like ^225Ac-DOTATATE are under investigation, potentially offering enhanced DNA damage with distinct toxicity profiles [120,121]. Future clinical trials focusing on combination strategies and biomarker-driven patient selection will be critical to integrate PRRT into personalized therapeutic algorithms effectively.

### 4.3. Targeted Agents and Molecular Pathways: Opportunities and Obstacles

Targeting intracellular signaling pathways implicated in pNET pathogenesis, notably the PI3K/AKT/mTOR cascade, has yielded clinically approved options such as everolimus [122]. While everolimus improves PFS in advanced non-functional pNETs, resistance mechanisms and toxicity limit its long-term impact [123]. Ongoing development of second-generation mTOR inhibitors and dual PI3K/mTOR inhibitors reflects efforts to enhance durability, though balancing efficacy and safety remains a challenge [124,125]. Rational combinations pairing mTOR inhibitors with anti-angiogenic agents or SSAs are under early clinical evaluation, yet definitive evidence for superiority is lacking. Anti-angiogenic TKIs, such as sunitinib and emerging agents like surufatinib and cabozantinib, demonstrate promising efficacy by targeting VEGFR and related kinases, with possible immunomodulatory benefits [126,127,128,129]. However, identifying biomarkers predictive of response and optimizing the combination with immunotherapy remains an unmet need.

### 4.4. Immunotherapy: Emerging Concepts and Clinical Challenges

Immune checkpoint inhibitors (ICIs) have transformed treatment paradigms across many cancers but have shown limited efficacy as monotherapy in well-differentiated pNETs, likely due to low tumor mutational burden and minimal PD-L1 expression [130,131,132]. Current research focuses on combinatorial approaches integrating ICIs with TKIs, PRRT, or chemotherapy to enhance immunogenicity and overcome immune resistance. Novel immunotherapies, including bispecific T-cell engagers, tumor-infiltrating lymphocytes (TILs), and CAR-T cells targeting neuroendocrine-specific antigens such as SSTR2 or CEACAM5, remain in preclinical or early clinical phases, necessitating further validation of safety and efficacy [133,134].

To better illustrate the current therapeutic landscape of pancreatic neuroendocrine tumors and the role of biomarkers in guiding treatment choices, we have summarized key information in two complementary tables. Table 3 provides a balanced overview of the advantages and challenges linked to these treatments, offering critical insight into their practical benefits and limitations. Table 4 outlines the main biomarkers and selection criteria associated with each therapy, highlighting their potential for personalization and important considerations for clinical application. Together, these tables help contextualize how molecular profiling and biomarker-driven strategies can inform more tailored and effective therapeutic approaches for patients with pNETs.

## 5. The Microbiome as a Gateway to Precision Medicine in Neuroendocrine Tumors

The gut microbiota plays a pivotal role in maintaining human health and has emerged as a key modulator of carcinogenesis and therapeutic outcomes through its influence on inflammatory processes and immune regulation. Its potential as both a biomarker and therapeutic target has been increasingly recognized, particularly in gastrointestinal adenocarcinomas. However, the role of the gut microbiota in GEP-NECs, and especially in pNETs, remains underexplored and insufficiently characterized [135]. Although current evidence is still limited, recent studies have begun to highlight a potential causal link between gut dysbiosis and GEP-NECs, suggesting that microbial communities and their metabolites may contribute to the tumor microenvironment and the pathogenesis of neuroendocrine tumors [136,137]. These early findings advocate for a paradigm shift where the gut microbiome is no longer considered a mere bystander but rather an active participant in tumor biology. The seminal work by Mohamed et al. [138] provided the first clinical insight into the altered microbial landscape in GEP-NECs patients, reporting not only a reduction in bacterial diversity and an increase in fungal species (notably *Candida*, *Ascomycota*, and *Saccharomycetes*) but also significant correlations between specific bacterial taxa (e.g., *Bacteroides fragilis*, *Eggerthella lenta*) and tumor grade, dietary patterns, and treatment response. These observations point toward the feasibility of using microbiota composition as a prognostic or predictive biomarker in GEP-NECs. More recently, a 2024 Mendelian randomization study by Zhang et al. [139] strengthened the hypothesis of a genetically inferred causal association between the gut microbiota and GEP-NECs. Specific taxa, such as *Negativicutes*, *Selenomonadales*, *Peptococcaceae*, *Candidatus Soleaferrea*, and *Desulfovibrio*, were associated with an increased risk for pNETs, while others like the *Bacteroidales S24-7 group* and *Eubacterium oxidoreducens group* appeared to exert protective effects. These findings open the door to microbiome-based risk stratification and even preventive interventions in genetically susceptible individuals. Nevertheless, further investigation is critically needed. Future studies should focus on validating these associations in larger and well-characterized pNET cohorts, integrating multi-omic approaches (metagenomics, metabolomics, transcriptomics) and exploring the mechanistic underpinnings of microbiome–host interactions in neuroendocrine tumorigenesis. Moreover, defining the dynamic interplay between the gut microbiota and therapeutic efficacy may reveal novel microbiota-targeted strategies, including dietary modulation, probiotics, prebiotics, or even fecal microbiota transplantation, as adjuvant tools to enhance treatment outcomes.

## 6. Conclusions

Despite considerable progress in molecular profiling, the clinical translation of emerging biomarkers in pNETs, such as ctDNA and microRNAs, remains constrained by limited validation, lack of standardization, and inconsistent clinical utility. Traditional markers like Ki-67, Chromogranin A, and somatostatin receptor expression still guide diagnosis and treatment, yet they fall short in capturing the biological complexity, intratumoral heterogeneity, and evolving resistance mechanisms that characterize pNETs. To achieve truly personalized therapy, the field must shift from static, descriptive biomarker panels toward dynamic and integrative models that inform treatment sequencing, predict response, and guide rational combination strategies. While targeted therapies and PRRT have improved clinical outcomes, durable responses are often undermined by both intrinsic and acquired resistance, underscoring the urgent need for more mechanistically informative biomarkers. A critical next step involves the rigorous clinical validation of novel molecular and functional biomarkers, supported by multi-omic platforms and AI-driven analytical tools capable of integrating complex datasets into actionable clinical algorithms. This transition will require robust interdisciplinary collaboration to optimize treatment efficacy while minimizing toxicity and preserving patient quality of life. In this evolving context, the gut microbiota has emerged as a promising and underexplored axis of tumor modulation in pNETs. Its role as both a biomarker of disease progression and a modifiable therapeutic target offers a unique opportunity to expand the toolbox of precision oncology. Incorporating microbiota-based strategies, alongside molecular and imaging biomarkers, could enable more holistic, adaptive, and personalized approaches to managing neuroendocrine neoplasms. Only through such systematic, cross-disciplinary innovation can personalized medicine in pNETs move from theoretical promise to clinical impact.

## Figures and Tables

**Table 1:** Advantages, challenges, and clinical readiness of established biomarkers in pNETs.

Biomarker	Advantages	Challenges	Clinical Readiness
Chromogranin A (CgA)	- Widely used for diagnosis, prognosis, and monitoring [57,58,59,64,65,66,67,68,70]. - Reflects tumor burden and secretory activity.	- Assay variability and lack of standardization. - False positives from benign conditions and PPI use. - Limited sensitivity in small or early tumors [63,64,65,66].	Established clinical biomarker; best used in combination with imaging and other tests [70].
Ki-67	- Standard for grading and prognostication [9,71,72,73,74,75,76,77,78,79,80,81,82]. - Guides treatment decisions. - Correlates with survival.	- Intratumoral heterogeneity. - Interobserver variability. - Manual counting affects reproducibility [79,80].	Gold standard histopathological marker; requires standardized assessment protocols [80,81].
SSTR2	- Central to imaging (^68^Ga-DOTATATE PET/CT) and targeted therapies [95,96,97,98,99,100,101,102,103,104,105,106,107,108]. - High sensitivity and specificity.	- Reduced sensitivity for lesions < 5 mm. - False negatives in SSTR-negative tumors. - Technical challenges [108].	Well-established for clinical use; crucial for staging and therapy planning.

**Table 2:** Advantages, challenges, and clinical readiness of emerging biomarkers in pNETs.

Biomarker	Advantages	Challenges	Clinical Readiness
ctDNA	- Non-invasive, dynamic tumor genotyping and monitoring [83,84,85,86]. - Captures genetic and epigenetic info. - Potential early detection of progression.	- Low levels in early/low-grade tumors. - Methodological variability and lack of standardization [89]. - Limited large-scale validation.	Promising but experimental; requires further validation and standardization [88,89].
miRNAs	- Stable in biofluids; non-invasive testing [90,109]. - Diagnostic and prognostic potential. - Can improve accuracy combined with CgA [91,92,93,94].	- Lack of methodological standardization. - Limited large-scale prospective validation. - Heterogeneous data [90,91,92,93,94,109].	Early clinical research stage; promising but needs robust validation and harmonization.

**Table 3:** Comparison of treatment options in pNETs: benefits and limitations.

Therapy	Advantages	Challenges
SSAs	Good tolerability, proven PFS benefit [67,108]	Limited curative potential, mainly disease control
PRRT	High response rates, personalized via imaging [114,116]	Toxicity risk, limited in low SSTR expression tumors [118]
mTOR inhibitors	Improved PFS, target key pathway [122]	Resistance and side effects limit efficacy [123]
Anti-angiogenic TKIs	Target tumor microenvironment, immunomodulatory [127,128,129,130,131]	Side effects, resistance
ICIs	Potential synergy with other treatments [133,134]	Low monotherapy efficacy, experimental combinations [130,131,132]

**Table 4:** Overview of therapeutic strategies in pNETs: biomarker-guided personalization and clinical insights.

Therapy	Biomarkers/Selection Criteria	Personalization Potential	Notes
SSAs	SSTR expression; hormone secretion profile [108,110]	Selection by receptor expression; hormone status	Standard first-line therapy
PRRT	High uptake on ^68Ga-DOTATATE PET/CT [116,120,121]	Theranostic approach enables patient-specific use	Requires functional imaging and renal function
mTOR inhibitors	Mutations in TSC2, PTEN, PIK3CA [19]	Potential for genomic-driven combinations	Resistance limits long-term efficacy
Anti-angiogenic TKIs	VEGF/VEGFR expression and angiogenic markers [125,126]	Emerging biomarkers to optimize therapy	Immunomodulatory effects under evaluation
ICIs	PD-L1 expression, tumor mutational burden [130,131,132]	Low predictive biomarkers; combos to enhance effect	Early-phase trials ongoing

## Data Availability

Not applicable.
